# Prevalence and risk factors of burnout among community pharmacists in the Aseer region, Saudi Arabia: a cross-sectional study

**DOI:** 10.3389/fphar.2026.1737667

**Published:** 2026-01-12

**Authors:** Moteb Khobrani, Sultan M. Alshahrani

**Affiliations:** Department of Clinical Pharmacy, College of Pharmacy, King Khalid University, Abha, Saudi Arabia

**Keywords:** burnout, mental fatigue, occupational health, occupational stress, pharmacists, Saudi Arabia, workload

## Abstract

**Background:**

Burnout incidents among community pharmacists continue to increase globally, resulting in impaired job performance and patient care as well as mental health deterioration. Research studies on pharmacist burnout and its connected risk factors remain insufficient for Saudi Arabian pharmacists.

**Objectives:**

This research aimed to investigate burnout frequency alongside related risk elements for Saudi Arabian community pharmacists in the Aseer region of Saudi Arabia.

**Materials and Methods:**

A cross-sectional survey was conducted among community pharmacists in the Aseer region of Saudi Arabia between March and July 2025. A structured questionnaire collected data on demographics, occupational factors, and burnout using the Maslach Burnout Inventory–Human Services Survey (MBI-HSS). Statistical analyses were performed using SPSS (version 25.0), applying chi-square tests, t-tests, Pearson correlations, and logistic regression at a significance level of p < 0.05.

**Result:**

The analysis revealed widespread burnout symptoms among study participants, including emotional exhaustion in 78.2% of respondents. In comparison, depersonalization affected 65.4% of participants, and a decline in personal accomplishment was experienced by 72.6%. Overall, a high proportion of participants demonstrated moderate-to-high burnout based on MBI-HSS cutoffs. Significant positive correlations were observed between burnout and heavy workload (p = 0.002), extended working hours (p = 0.004), and insufficient financial compensation (p = 0.006). Female pharmacists showed higher emotional exhaustion scores than their male counterparts, with 29.8 ± 6.4 and 25.6 ± 5.9, respectively (p = 0.03). Pharmacists who exceeded 48 h per week showed higher levels of professional burnout (p = 0.01), while those younger than 35 years demonstrated increased emotional exhaustion compared to their older counterparts (p = 0.02).

**Conclusion:**

The findings indicate a high prevalence of burnout among community pharmacists in the Aseer region of Saudi Arabia, influenced by workload, long working hours, insufficient compensation, and demographic factors. Implementation of workload management systems, mental health support, and career development resources is recommended to decrease burnout and enhance pharmacist well‐being and patient care quality.

## 1 Introduction

Burnout represents a work-related psychological condition that arises from prolonged workplace stress consisting of emotional exhaustion, depersonalization, and reduced personal accomplishment (Van Dam, 2021). Numerous health organizations identify burnout as an occupational health issue that affects personnel in high-stress areas, particularly healthcare (Edú-Valsania et al., 2022). The World Health Organization (WHO), through its International Classification of Diseases (ICD-11), classifies burnout as a work-related occupational phenomenon rather than a medical diagnosis, which can threaten employee wellness, business performance, and efficiency (Hualparuca-Olivera and Betalleluz Palomino, 2023). Burnout is a structural problem that impacts professionals' efficiency, workplace dynamics, and patient care standards (de Beer, 2021).

Healthcare professionals experience significant consequences due to burnout in their professional lives. Healthcare staff, including physicians, nurses, and pharmacists, are at increased risk because of demanding work environments characterized by long working hours, emotionally intensive patient interactions, administrative requirements, and rising performance expectations, which collectively contribute to mental exhaustion and stress (Eltorki et al., 2022; Sibeoni et al., 2021; Taranu et al., 2022). Prior research has demonstrated that healthcare burnout is associated with increased medical errors, reduced patient satisfaction, and higher staff turnover rates (Li et al., 2023; Owoc et al., 2021). Among pharmacists, burnout further compromises medication safety, leading to prescription errors, inappropriate dosing, and suboptimal patient counseling (Bradley et al., 2024; Patel and Andy, 2021).

Pharmacists encounter distinct occupational challenges that put them at high risk of experiencing burnout (Wash et al., 2024). Community pharmacists often operate independently, without the multidisciplinary support structures commonly available in hospital settings, while managing ongoing patient care and complex medication responsibilities (Bradley et al., 2024; Rushworth et al., 2024). In recent years, expanding professional roles including chronic disease management, immunization services, and public health engagement have further increased workload demands, heightening vulnerability to burnout when organizational support is insufficient (Gysel and Tsuyuki, 2024; Strand, 2025; Jarab et al., 2024). Recent studies show that pharmacist burnout ranges between 52% and 90%, with frontline community pharmacists being most affected because of limited organizational support (Borowitz et al., 2024; Martello et al., 2024). A recent systematic review further highlights that workplace stressors, evolving pharmacy roles, and inadequate organizational support are key workforce pressures that contribute directly to pharmacist burnout across diverse international settings. (Barakat et al., 2025).

The negative outcomes of pharmacist burnout extend beyond individual wellbeing to organizational performance. Burnout contributes to job dissatisfaction, absenteeism, and turnover, which in turn affect workforce stability and patient safety (Ivanova et al., 2024; Walker et al., 2025; Arefin and Global Health Institute Research Team, 2025; Grotowska et al., 2025). Pharmacists who work excessive hours or experience constant emotional strain are more likely to make medication errors and communicate less effectively with patients (Alhomoud and Alrasheedy, 2024).

In Saudi Arabia, healthcare employee burnout has gained increasing attention amid rapid health sector transformation under Vision 2030 (Alqarni et al., 2022; Siraj et al., 2023; Suleiman and Ming, 2025). While reforms aim to enhance service quality and access through public–private collaboration (Mani and Goniewicz, 2024), they have concurrently intensified workloads and administrative demands on healthcare professionals, including pharmacists (Al Khashan et al., 2021; Almogbel, 2021). The expansion of pharmaceutical services, increasing patient volumes, and growing expectations for pharmacists’ clinical involvement have collectively amplified occupational stress, contributing to rising burnout levels (Almogbel, 2021).

Within Saudi community pharmacy settings, excessive workload, extended shifts, and staff shortages remain persistent stressors (Alnezary et al., 2024; Alqassab et al., 2024; Bounthavong, 2024). Many pharmacists work prolonged hours, frequently as the sole practitioner, which limits recovery time and increases psychological strain. Burnout in this context is further exacerbated by emotionally demanding patient interactions involving complex health conditions, medication adverse effects, affordability concerns, and insurance-related disputes (Younes et al., 2024; Alemede et al., 2024; Alshorman et al., 2024).

Freudenberger initially described burnout in the 1970s, and Maslach and Jackson later refined it using the Maslach Burnout Inventory (MBI) (Franco-Paredes and Tuells, 2023; Avila et al., 2021). MBI serves as a widely implemented assessment tool to measure burnout through its three dimensions: emotional exhaustion, depersonalization, and personal accomplishment (Schommer et al., 2022). This study was guided by the Job Demands-Resources Model, which proposes that high job demands (e.g., workload, extended hours) and limited resources (e.g., low pay, inadequate support) interact to produce burnout.

Burnout affects pharmacists not only at an individual level but also undermines the healthcare system. High burnout levels reduce patient trust, weaken service quality, and contribute to workforce instability, ultimately threatening healthcare sustainability (Santos et al., 2022; Dee et al., 2023; Agata et al., 2023; Sami et al., 2021). International evidence from North America, Europe, and the Middle East consistently reports burnout prevalence exceeding 50%, with community pharmacists experiencing the highest risk due to sustained workload and administrative burden (Schwerdtfeger et al., 2024).

During the COVID-19 pandemic, burnout among pharmacists reached 70%–80%, with elevated emotional exhaustion and depersonalization (Alhomoud and Alrasheedy, 2024; Bustamante Izquierdo et al., 2024; Mohammed et al., 2025; Kiriazopoulos et al., 2025). Regional studies from the Middle East, including studies from Lebanon, Qatar, and Abu Dhabi, similarly reported workload and work–life imbalance as dominant burnout drivers, underscoring shared structural stressors while highlighting the relevance of organizational support within regional contexts (Mohammed et al., 2025; Kiriazopoulos et al., 2025).

Several interventions, such as improved staffing, peer-support programs, and mindfulness-based approaches, have been suggested to mitigate burnout, yet their effectiveness within Saudi community settings remains underexplored (Forehand et al., 2022; Deniz and Eren, 2024; Barnett et al., 2022; Potter and Cadiz, 2021; Arhabal, 2024; Fendel et al., 2021; Alshammari et al., 2024). Despite the recognition of pharmacist burnout, few Saudi studies have quantitatively assessed its prevalence or explored its determinants at the regional level (Alwhaibi et al., 2022; Maslach et al., 1997).

Despite increasing recognition of pharmacist burnout in Saudi Arabia, no studies have quantified its prevalence or identified associated factors among community pharmacists in the Aseer region. This study addresses that gap by examining the prevalence and key risk factors of burnout among community pharmacists in Aseer, thereby contributing evidence to inform targeted wellbeing interventions and improve pharmaceutical care quality.

## 2 Materials and methods

### 2.1 Study design

The study consists of a cross-sectional survey, conducted between March and July 2025, to determine the prevalence of burnout and related occupational risks among community pharmacists in Aseer, Saudi Arabia. The study design enabled a one-time burnout measurement to help discover work-related and demographic determinants that lead to burnout.

### 2.2 Data collection and survey

The survey was distributed electronically via Google Forms. Stratification was based on pharmacy type (chain vs. independent) and geographic location within the Aseer region. Strata sizes were determined based on the estimated distribution of community pharmacies within the region, and proportional allocation was applied to reflect the relative size of each stratum. Participation was voluntary, and responses were collected anonymously. Incomplete responses were excluded from analysis (n = 18), ensuring only complete datasets were analyzed.

Given that the study employed the Maslach Burnout Inventory–Human Services Survey (MBI-HSS), a well-established and extensively validated instrument, and that the target population consisted of licensed community pharmacists, formal pilot testing was not deemed necessary. The questionnaire items were used as originally designed without modification, thereby preserving their validated structure and content validity.

The data collection process used structured questionnaires to assess four aspects: demographics and occupational details, burnout evaluations, burnout threats, and their effects on professional achievement. The demographic part included critical variables: age, gender, marital status, educational background, and professional years of experience. Occupational variables included the distinction of pharmacy practice as independent or chain, the number of weekly work hours, the number of patients cared for, and the self-assessed workload intensity.

Healthcare professionals underwent burnout assessment through the widely trusted Maslach burnout Inventory-Human Services Survey (MBI-HSS). The MBI-HSS consists of 22 items organized into three distinct theoretical sections, which include Emotional Exhaustion (EE) and Depersonalization (DP), as well as Personal Accomplishment (PA). Topic models included EE, which had nine items to detect stress levels and work burnout; DP, which had five items to monitor detached behavior from patients; and PA, which had eight items to measure work competency evaluation. The participants used a seven-point Likert scale to rate their responses from “Never” (0) to “Everyday” (6), while higher scores for EE and DP and lower scores for PA indicated higher levels of burnout.

Before starting the survey, participants were provided with a brief standardized explanation defining Emotional Exhaustion, Depersonalization, and Personal Accomplishment, adapted from the MBI-HSS manual, to promote consistent interpretation of these constructs across respondents.

### 2.3 Reliability and validity

The MBI-HSS proved its validity through various assessments with community pharmacists across Vietnam and three Mediterranean countries: Jordan, Greece, and Qatar. The reliability of the instrument was tested through Cronbach’s alpha to evaluate internal consistency across the subscales.

Burnout levels were categorized according to the cutoff criteria recommended in the Maslach Burnout Inventory–Human Services Survey manual (Franco-Paredes and Tuells, 2023). Participants with emotional exhaustion (EE ≥ 27), depersonalization (DP ≥ 10), and personal accomplishment (PA ≤ 33) were classified as having high EE, high DP, and low PA, respectively. These thresholds have been consistently applied in healthcare burnout studies, including among pharmacists, and were used in this study to determine the prevalence rates for each burnout domain.

### 2.4 Sample size calculation

The following formula was applied to measure the sample size for assessing proportions:

$$n=\left(Z^{2}*P*\left(1-P\right)\right)/E^{2}$$

Where Z = 1.96 for a 95% confidence level, P = 0.43 (estimated prevalence of burnout based on previous studies in similar settings) (10), E = 0.05 (error margin). Replacing these values:

$$n=\left(1.96\right)2\cdot 0.43\cdot \left(1-0.43\right)/\left(0.05\right)2\approx 376$$

Due to limited response rates and challenges in reaching pharmacists in remote areas, the final achieved sample size was 284, representing a response rate of approximately 75.5%. Post-hoc power estimation indicated that the achieved sample size retained acceptable statistical power (>80%) for detecting moderate associations between burnout outcomes and key occupational variables, while balancing feasibility constraints related to participant availability and regional access. This number ensured sufficient statistical power while maintaining feasibility for recruitment.

### 2.5 Sampling and recruitment

Participants involved community pharmacists in various settings across the Aseer region, Saudi Arabia, including chain and independent pharmacies. A stratified sampling technique was employed to ensure representation from both urban and rural areas, as well as across different pharmacy types. Age-based stratification was not applied during sampling, as recruitment relied on voluntary participation; therefore, the observed age distribution reflects the actual workforce availability and response patterns within community pharmacies in the region, where younger pharmacists constitute a larger proportion of the active workforce. Eligible participants were licensed community pharmacists who had been working in their role for at least 1 year. Pharmacists working exclusively in managerial roles or academia were excluded to maintain the focus on direct patient care settings.

### 2.6 Statistical analysis

Statistical analysis was conducted utilizing SPSS version 25. Descriptive statistics, such as frequency, percentage, mean, and standard deviation, were used to summarize demographic and occupational variables and burnout scores. Inferential analysis was employed to explore associations between burnout and various factors. Chi-square tests assessed the relationships between categorical variables, such as gender and depersonalization levels. Independent t-tests and ANOVA compared mean burnout scores across demographic and occupational subgroups. Pearson’s correlation coefficient evaluated the strength and direction of relationships between continuous variables, such as weekly working hours and emotional exhaustion scores. Logistic regression analysis was executed to identify predictors of high burnout, incorporating independent variables such as workload, patient volume, and access to workplace support. Potential confounders, including gender, age, and years of experience, were controlled for in logistic regression analysis to minimize bias. Reliability analysis was conducted utilizing Cronbach’s alpha, with values above the acceptable threshold of 0.70 across emotional tiredness, depersonalization, and personal accomplishment subscales to ensure the internal consistency of the burnout dimensions. Statistical significance was recognized at p < 0.05.

### 2.7 Ethical considerations

The Research Ethics Committee at King Khalid University (ECM#2025-401) approved the study. Detailed knowledge regarding the study’s objectives, procedures, and confidentiality measures was given to the participants. Proper consent was obtained from each participant before the survey started. Responses were anonymized, and all data were handled securely to ensure privacy. The study followed the guidelines of the Declaration of Helsinki for ethical research, including the participation of human participants.

## 3 Results

### 3.1 Participant demographic and occupational profile

The study included 284 community pharmacists from the Aseer region of Saudi Arabia, representing one of the first regional datasets examining burnout among community pharmacists in this area. The demographic and occupational characteristics of participants are summarized in Table 1. The males accounted for 79.0%, and the females accounted for 21.0%. Most pharmacists (47.5%) were aged between 20 and 29 years, followed by 43.0% aged 30–39. About 7.0% of pharmacists were observed to be between the age group 40–50 years. The oldest age group observed was 50 years and above, accounting for 2.5%. Regarding marital status, 68.0% of the participants were single, while 32.0% were married. Professional experience varied, with 46.0% having 1–5 years of experience, 45.8% having 6–10 years, and only 8.2% having more than 10 years of experience. Educational qualifications showed that 76.1% held a bachelor’s degree in pharmacy (BSc Pharm), 17.3% held a PharmD, and 6.6% had postgraduate degrees (Master’s or PhD). Most pharmacists worked in chain pharmacies (77.1%), while the remaining 22.9% worked in independent pharmacies.

**TABLE 1:** Demographic and occupational characteristics of participants (N = 284).

Characteristic	n	%
Gender		
Male	224	79.0
Female	60	21.0
Age group (years)		
20–29	135	47.5
30–39	122	43.0
40–49	20	7.0
≥50	7	2.5
Marital status		
Single	193	68.0
Married	91	32.0
Years of experience		
1–5	131	46.0
6–10	130	45.8
>10	23	8.2
Educational qualification		
PharmD	216	76.1
Bachelor’s degree (BSc. Pharm)	49	17.3
Postgraduate (Master’s, PhD)	19	6.6
Type of pharmacy		
Chain	219	77.1
Independent	65	22.9
Hours worked per week		
≤20	19	6.7
21–40	131	46.1
41–60	101	35.6
>60	33	11.6
Shifts worked per day		
One	95	33.5
Two	144	50.7
Three	45	15.8
Patients served per day		
<20	30	10.6
21–50	119	41.9
51–100	102	35.9
>100	33	11.6

This distribution reflects the dominant presence of chain pharmacies in Aseer and provides a useful context for understanding burnout risk patterns in modern Saudi community pharmacy settings.

Occupational data revealed that 46.1% of participants worked 21–40 h per week, 35.6% worked 41–60 h, and 11.6% worked more than 60 h weekly. Approximately half of the pharmacists (50.7%) worked two shifts per day, while 33.5% worked one shift, and 15.8% worked three shifts daily. Patient volume varied significantly, with 41.9% serving 21–50 patients daily, 35.9% serving 51–100 patients daily, and 11.6% serving over 100 patients daily.

### 3.2 Occupational workload and burnout scores

MBI-HSS was used to measure burnout, as shown in Table 2. The mean emotional exhaustion (EE) score was 32.4 (SD = 8.7), indicating moderate to high levels of emotional fatigue. Depersonalization (DP) scores averaged 14.6 (SD = 4.3), reflecting moderate levels of detachment and cynicism. Personal accomplishment (PA) scores averaged 28.9 (SD = 6.5), signifying a reduced sense of professional efficacy. The internal consistency of the MBI-HSS subscales was confirmed, with Cronbach’s alpha values of 0.91, 0.79, and 0.84 for EE, DP, and PA, respectively.

**TABLE 2:** Burnout scores by maslach burnout inventory-human services survey subscales (N = 284).

Subscale	Mean (SD)	Cronbach’s alpha
Emotional exhaustion (EE)	32.4 (8.7)	0.91
Depersonalization (DP)	14.6 (4.3)	0.79
Personal accomplishment (PA)	28.9 (6.5)	0.84

### 3.3 Relationship between burnout and demographic and occupational factors

The relationships between burnout and demographic or occupational variables are shown in Table 3. Female pharmacists exhibited significantly higher DP scores than their male counterparts (p = 0.032). Younger pharmacists aged 20–29 were more likely to experience high EE (p = 0.028) than older age groups. Pharmacists with less experience (one to five years) had the highest burnout levels across all subscales (p = 0.044). Those working in chain pharmacies reported significantly higher burnout scores, particularly for EE and DP (p = 0.027). Additionally, pharmacists working over 60 h weekly or serving more than 50 patients daily exhibited significantly higher EE and DP scores than those working fewer hours or serving fewer patients (p = 0.016 and p = 0.021, respectively).

**TABLE 3:** Association between demographics, occupational variables, and burnout.

Variable	High EE (%)	High DP (%)	Low PA (%)	p-value
Gender				0.032*
Male	68.8	55.4	72.3	
Female	31.2	44.6	27.7	
Age group				0.028*
20–29	49.5	41.0	62.0	
30–39	38.3	39.7	28.4	
>40	12.2	19.3	9.6	
Years of experience				0.044*
1–5	52.6	40.3	64.5	
6–10	28.6	33.0	22.9	
>10	18.8	26.7	12.6	
Type of pharmacy				0.027*
Chain	67.2	60.4	71.5	
Independent	32.8	39.6	28.5	
Hours worked per week				0.016*
≤40	29.5	25.2	23.4	
41–60	43.3	42.5	48.7	
>60	27.2	32.3	27.9	
Patients served daily				0.021*
<20	10.4	15.6	12.3	
21–50	38.9	32.0	35.8	
51–100	37.5	39.1	43.0	
>100	13.2	13.3	8.9	

* Statistically significant p-value <0.05.

EE: emotional exhaustion, DP: depersonalization, PA: personal accomplishment.

## 4 Discussion

Community pharmacist burnout shows increasing concern due to high workloads, including job stress factors and emotional exhaustion (Cline and Mehta, 2022). The study analyzes burnout occurrence and risk elements among pharmacists in Saudi Arabia. Identifying these elements is essential to developing interventions to boost patient satisfaction and healthcare. This study revealed a notable presence of burnout among community pharmacists in the Aseer region, reflecting the growing strain associated with increased service demands and limited workforce support. The findings reaffirm that burnout among pharmacists is not only a workplace issue but also an emerging public health concern.

The studies conducted in different regions demonstrate that excessive workload and emotional exhaustion cause extensive burnout. In this study, excessive workload refers to prolonged working hours, high patient volume, and the accumulation of administrative and clinical responsibilities beyond reasonable capacity, while extensive or substantial burnout denotes persistently elevated levels of emotional exhaustion and depersonalization accompanied by reduced personal accomplishment. Pharmacists in Saudi Arabia share similar burnout experiences because of prolonged working hours, administrative responsibilities, and patient interactions (Almogbel, 2021). The study by Weichel et al. suggested that pharmacist burnout exists in multiple healthcare environments. However, it is more common in community pharmacies because patients and business requirements generate stressful conditions (Weichel et al., 2021). Community pharmacists in Saudi Arabia experience prolonged and demanding work, creating emotional exhaustion and depriving them of personalized connections with patients (Aldaiji et al., 2022). This observation is consistent with the present findings, where workload intensity and role pressure appeared to be among the most influential factors contributing to burnout. Societal norms within the Saudi context, including expectations of extended availability, limited flexibility in work schedules, and strong professional obligations toward patient service, may further intensify workload pressure and contribute to burnout. The results show that factors, including workforce shortages and societal norms, contribute to pharmacist burnout in Saudi Arabia.

Several studies reported that pharmacist burnout directly correlates with elevated medication errors, reduced patient care standards, and mental fatigue (Chong et al., 2022; Fadare et al.; Trinh et al., 2025). The current study’s findings agree with the results by illustrating that burnout negatively impacts pharmacist performance. According to the survey, workplace support is not sufficient to reduce burnout. However, Rogozinska suggested organizational support and workplace efforts as potential solutions to burnout. Burnout exists globally, and solutions must align with specific work environments and cultural norms in different regions (Rogozińska-Pawełczyk, 2024). The study also explored financial compensation as a key element for pharmacist burnout, and there have not been enough relevant studies in the past. A European study by Todorova et al. showed workload and job satisfaction were the main burnout factors, but financial encouragement remained unimportant in their analysis (Todorova et al., 2024). In contrast, the present findings emphasize financial stress as a stronger determinant within the Saudi context, where remuneration structures and job growth opportunities may not align with increasing professional responsibilities. Inadequate financial compensation emerged as a primary stressor for Saudi pharmacists due to workload and minimal salary growth opportunities, particularly in busy community pharmacies. The finding indicates that economic factors create substantial burnout when pharmacists receive wages below the evolving professional demands (Almogbel, 2021; Al-Jumaili et al., 2023). This observation aligns with global evidence demonstrating that inadequate earnings, limited employment benefits, and ongoing financial pressures significantly contribute to pharmacist burnout and workforce instability. (Barakat et al., 2025).

Numerous studies suggest that the COVID-19 era has worsened the workload and stress among pharmacists worldwide. The pandemic caused pharmacists to experience greater work demands (Bhamra et al., 2021; Hedima et al., 2022; Mohammad et al., 2022). According to the findings, there were increased COVID-19 cases but fewer pharmaceutical resources, along with heightened dangerous health conditions. Saudi pharmacists received minimal support from their institutions, which increased their stress levels (Aljadeed et al., 2021). Research findings show that burnout among Saudi healthcare personnel has reached significant levels during COVID-19, reaching an estimated rate of 75% among professionals (Alanazi et al., 2021; Aljuffali et al., 2022). Pharmacists experience exceptionally severe burnout levels because of extended work shifts, insufficient staffing, and mounting pressure from patients (Johnston et al., 2023). Community pharmacists face extensive psychological fatigue and detachment from their patients due to long working hours and numerous patient interactions (Alhomoud and Alrasheedy, 2024). Survey results using the Copenhagen Burnout Inventory revealed that more than 80% of Saudi community pharmacists documented personal and work-related burnout (Alhomoud and Alrasheedy, 2024; Youssef et al., 2022). The persistence of similar burnout patterns in this post-pandemic period suggests that systemic workforce challenges, rather than pandemic-specific pressures alone, continue to drive stress and exhaustion among community pharmacists. The present study did not specifically stratify participants based on employment timing relative to the COVID-19 pandemic; however, the persistence of similar burnout patterns in this post-pandemic period suggests that ongoing structural and workforce challenges, rather than pandemic-related factors alone, continue to drive pharmacist stress and exhaustion.

Furthermore, the present study makes a distinctive contribution to burnout findings by highlighting how local workplace environments impact pharmacist stress levels in Saudi Arabia. The conclusions of this study differ from those of Western countries in that Saudi pharmacists encounter substantial barriers to mental health assistance. Working environments in Saudi Arabia lack mental health programs, with minimal advancement opportunities and traditional expectations, generating unique challenges regarding burnout. This interpretation aligns with the Job Demands–Resources (JD-R) framework, which explains how high work demands combined with limited institutional support and recognition can progressively lead to emotional exhaustion and reduced professional accomplishment. By situating these findings within Saudi Arabia’s Vision 2030 transformation, this study highlights how the rapid expansion of private community pharmacy services has increased attention toward workforce wellbeing, resilience, and equitable professional development.

The findings reinforce that burnout among community pharmacists in Saudi Arabia is influenced by a combination of occupational demands and contextual factors, emphasizing the importance of continued attention to pharmacist wellbeing within the evolving healthcare landscape.

## 5 Strengths and limitations of the study

The strength of this study lies in its focus on community pharmacists within the Aseer region of Saudi Arabia, which provides important regional evidence on burnout in a group that has received limited attention in previous research. The study design allowed for the inclusion of participants from different demographic categories and workplace settings, enabling a clearer understanding of how factors such as gender, work experience, and pharmacy type relate to burnout dimensions. To the best of our knowledge, this is the first study to specifically assess burnout and its associated risk factors among community pharmacists in the Aseer region, contributing valuable local data to the growing national and global discussion on pharmacist wellbeing.

However, the study also has several limitations. It relies on self-reported data, which may introduce recall or social desirability bias and influence the accuracy of participants’ responses. Another limitation is the cross-sectional design, which cannot determine causal relationships between the identified variables. Thus, we cannot conclude whether workplace stressors lead to burnout or whether existing burnout intensifies perceptions of workload and dissatisfaction. This question remains open and highlights the need for longitudinal and interventional studies to further explore these associations within the Saudi pharmacy context. In addition, although participants represented a range of age groups, older pharmacists were underrepresented, which may limit the ability to explore potential generational differences in burnout perceptions and experiences. Future studies employing age-stratified sampling may provide deeper insight into how burnout varies across different career stages. The achieved sample size, while sufficient for detecting moderate associations, did not reach the initially calculated target, which may have reduced the precision of some estimates. Finally, since the study included only community pharmacists, the findings may not be generalizable to other pharmacy sectors, such as hospital or academic settings.

## 6 Conclusion

The study indicates that burnout is highly prevalent among community pharmacists practicing in the Aseer region of Saudi Arabia, with a substantial proportion of participants demonstrating moderate-to-high burnout across emotional exhaustion, depersonalization, and reduced personal accomplishment domains, primarily due to heavy workloads, longer working hours, insufficient financial rewards, and hindered career pathways. Cultural and organizational factors, along with inadequate institutional support, are emphasized as key contributors to burnout. These findings highlight the need for focused workplace strategies that promote balanced workloads, adequate compensation, and mental health support. Future research should prioritize longitudinal designs to clarify causal relationships, evaluate the effectiveness of workplace and policy-level interventions, and assess the impact of organizational reforms on burnout trajectories over time. In addition, intervention-based studies examining staffing models, workload redistribution, and structured mental health programs are warranted to prevent pharmacist burnout and enhance job satisfaction, resulting in high-quality healthcare services and better patient outcomes. There remains a need to examine how organizational initiatives and policy-level strategies can mitigate burnout and strengthen pharmacist wellbeing across different regions of Saudi Arabia. Expanding this work to include other pharmacy sectors and geographical areas would also allow broader generalization and greater relevance for national workforce planning.

## Data Availability

The original contributions presented in the study are included in the article/supplementary material, further inquiries can be directed to the corresponding author.
